# Generation climate crisis, COVID-19, and Russia–Ukraine-War: global crises and mental health in adolescents

**DOI:** 10.1007/s00787-023-02300-x

**Published:** 2023-10-09

**Authors:** Johanna Lass-Hennemann, M. Roxanne Sopp, Norma Ruf, Monika Equit, Sarah K. Schäfer, Benedikt E. Wirth, Tanja Michael

**Affiliations:** 1 Division of Clinical Psychology and Psychotherapy, Saarland University, Saarbrücken, Germany; 2 Division of Clinical Child and Adolescent Psychology and Psychotherapy, Technische Universität Braunschweig, Braunschweig, Germany; 3 Department of Cognitive Assistants, German Research Center for Artificial Intelligence (DFKI), Saarbrücken, Germany; 4 Division of Clinical Psychology and Psychotherapy, Department of Psychology, Saarland University, Campus A1 3, 66123 Saarbrücken, Germany; 5 Leibniz Institute for Resilience Research, Mainz, Germany

**Keywords:** COVID-19, Russia-Ukraine-War, Climate change, Depression, Anxiety, Adolescence

## Abstract

Climate change, COVID-19, and the Russia-Ukraine War are some of the great challenges of our time. These global crises affect young people in a particularly vulnerable phase of their lives. The current study aimed to assess the impact of these crises on mental health (depression, anxiety, and health-related quality of life) in secondary school students in Germany. Furthermore, we assessed known predictors of mental health, such as socio-economic factors, individual life stressors, and resilience factors (self-efficacy, expressive flexibility) as covariates. In our sample of 3998 pupils, pandemic- and climate-related distress were linked to greater depression and anxiety and reduced health-related quality of life. War-related distress was associated with greater anxiety. Critically, these associations remained significant after controlling for all covariates, supporting the incremental predictive value of the crises measures. The study reveals a significant impact of the crises on the mental health of the current generation of adolescents. As such it suggests that mental health policies should include interventions that help youth to cope with the stress caused by the crises.

**Supplementary Information:**

The online version contains supplementary material available at 10.1007/s00787-023-02300-x.

## Introduction

Failure to mitigate climate change and failure of climate change adaptation, as well as interstate conflicts are particularly severe global risks in the short and long term [1]. Rapid global warming [2] and the outbreak of the Russia-Ukraine War (RUW) while the Coronavirus disease 2019 (COVID-19) pandemic was still considered a global health emergency mean that this decade is already considered to be particularly crisis-ridden. Young people are particularly affected by the consequences of these crises. First, global crises have a greater impact on young people because the crises are related to greater and more protracted uncertainties in young people’s futures. Second, young people face these stressors at a particularly vulnerable phase of their lives. Epidemiological studies show that the period of adolescence coincides with a sharp increase in mental health problems [3]. As mental health is a product of individual, social, and environmental factors [4], one may assume that global crises negatively impact adolescents’ mental health. With respect to COVID-19, numerous studies show that the pandemic had a profound negative effect on children and adolescents [5–8].

The impact of the other crises is less well researched, but surveys demonstrate that adolescents are fearful because of climate change. For example, a representative online study (*N* = 2001) of German adolescents between 14 and 17 years reveals that only 5% are not scared of the consequences of climate change, with 37% experiencing strong anxiety [9]. Tentative evidence links such fears to poor mental health in adolescents, particularly to anxiety and depression [10–12]. Similarly, preliminary evidence from the Netherlands and Central Europe suggests that the RUW has a negative impact on the mental well-being of adolescents not directly affected by the war [13, 14].

In sum, there is good evidence that COVID-19 has negative mental health consequences for adolescents and preliminary evidence that the climate crisis and the RUW are detrimental to their mental health. To evaluate the relative contribution and importance of these stressors on adolescents’ mental health, it is, however, necessary to examine them simultaneously. Up to date, no such study exists. In adults, two studies assessing worry about COVID-19, the climate crisis and the RUW on psychological well-being have been conducted. A survey study in Germany found that while participants ranked the RUW and climate change as the most worrying stressors, only COVID-19-related stress negatively predicted mental health [15]. In contrast, an Italian study found significant associations between all three crises and indicators of mental health [16].

When examining how potential stressors affect mental health, it is important to also consider potential protective factors. In fact, several resilience factors have been linked to coping with COVID-19. The present study focuses on two well-known resilience factors: self-efficacy and expressive flexibility. Self-efficacy describes the personal belief in being able to face new situations, difficulties and challenges [17] and is considered to be particularly relevant for dealing with global crises. It was established as a protective factor for adolescents during the COVID-19 pandemic [18]. Moreover, a negative association has been found between climate anxiety and self-efficacy [19].

Expressive flexibility is defined as the ability to adapt to changing contextual demands by enhancing and suppressing one’s one emotional responses [20] and may thus significantly contribute to coping with long-term stressors such as COVID-19 [21–23]. The concept has received particular attention in adolescents [24–26] with studies showing that high expressive flexibility is associated with fewer negative psychological consequences of trauma [27, 28]. However, to date there are no studies investigating the association between expressive flexibility and distress related to COVID-19, the climate crisis, and the RUW in adolescents.

The present study aims to assess the relative burden of the climate crisis, the COVID-19 pandemic, and the RUW on mental health (depression, anxiety, health-related quality of life) in a sample of 3998 adolescents while controlling for several risk and resilience factors (self-efficacy and expressive flexibility).

## Methods

### Study design

The study was conducted in Saarland, which is a federal state of Germany, between May and October 2022. It was approved by the local ethics committee and the local ministry of education.

### Sample

Our target sample was all 7 to 9th graders of all secondary schools in the Saarland (*N* = 98). In Germany, the majority of pupils in grades 7–9 are 12–16 years old. The final sample consisted of 3998 pupils from 58 different schools.

### Procedure

#### School recruitment

Schools were invited to participate by contacting each school’s principal via Email, providing information about the study. Details about onsite assessment are provided in the Supplementary Information.

#### Measures

The questionnaire package consisted of four parts: (1) Sociodemographic information, (2) crisis-related distress, (3) psychopathological symptoms and (4) resilience factors. Detailed information can be found in the Supplementary Information.

#### Sociodemographic data

The survey included questions on age, gender, and migration background. Subjectively experienced socio-economic status was measured using the German version of the MacArthur scale asking respondents to place themselves on a 10-steps “social ladder” [29].

#### Crisis-related distress

Pandemic-related, RUW-related, and climate-related distress were assessed with self-developed questionnaires. As a basis for the questionnaires, we used previously established questionnaires on climate anxiety [10], fear of war [30] and burden during the COVID-19 pandemic [7]. The self-developed questionnaires consisted of the same questions for each crisis, which were answered on a 5-point Likert scale.

#### Psychopathological symptoms

The patient health questionnaire-9 (modified for adolescents) [31] and the generalized anxiety subscale of the German version of the screen for child anxiety-related disorders [32] were used to assess depression and anxiety. The KIDSCREEN-10 Index [33] was used to assess Health-related quality of life (HRQol).

#### Resilience factors

The general self-efficacy short scale [34], and the child and adolescent flexible expressiveness Scale (CAFE; [25])^1^ were used to assess resilience factors.

### Data analyses

A series of multilevel models was fit to investigate associations between crisis-related distress and psychopathological symptoms. The acquired data were nested in a three-level structure, such that pupils (Level 1) were nested in classes (Level 2), which were nested in schools (Level 3). To test our hypotheses, we assessed whether introducing crisis-related distress as fixed effects improved model fit (Baseline model). Thereafter, we tested whether introducing random slopes for these crisis-related distress indices improved model fit. In the third step, we added sociodemographic characteristics as predictors and evaluated model improvement. Thereafter, we introduced individual stressor-related distress as a fixed effect and evaluated model fit. Finally, we introduced resilience factors as fixed effects. The model comprising the maximum number of fixed effects was used to evaluate the incremental validity of crisis-related distress in predicting psychopathological symptoms. To this end, we calculated standardized regression weights. Overall model prediction was assessed by calculating marginal and conditional R^2^. Models were fit using restricted maximum likelihood estimation and the lme4 package [35] in R [36]. All predictors were group-mean centered on class level [37]. The two-sided α level was set to 0.05 for all analyses. Degrees of freedom varied across analyses due to missing data.

## Results

### Sample characteristics

Sample characteristics as well as means and standard deviations of study variables are reported in Table 1.

**Table 1:** Descriptive statistics

Variable	*N*	Mean	SD	Min	Max
Sex ♀: 2275/**♂**: 1635/Diverse: 76	3986				
Age	3991	14.15	1.01	10	18
SES	3887	6.25	2.04	1	10
Climate—Subj. distress	3980	1.51	1.02	0	4
Climate—Sadness	3976	1.04	1.08	0	4
Climate—Helplessness	3962	0.90	1.14	0	4
Climate—Anxiety	3971	1.18	1.18	0	4
Climate—Anger	3960	1.16	1.27	0	4
Climate—Guilt	3964	0.99	1.06	0	4
Climate—Desperation	3963	0.92	1.12	0	4
Climate—Impact on psychosocial functioning	3983	1.91	0.96	1	5
War—Subj. distress	3964	1.81	1.09	0	4
War—Sadness	3973	1.68	1.23	0	4
War—Helplessness	3961	1.01	1.19	0	4
War—Anxiety	3969	1.66	1.28	0	4
War—Anger	3959	1.83	1.42	0	4
War—Guilt	3961	0.24	0.65	0	4
War—Desperation	3964	1.03	1.14	0	4
War—Impact on psychosocial functioning	3973	1.02	1.05	0	4
Pandemic—Subj. distress	3981	2.60	1.21	0	4
Pandemic—Sadness	3978	1.67	1.35	0	4
Pandemic—Helplessness	3968	1.50	1.38	0	4
Pandemic—Anxiety	3967	1.36	1.31	0	4
Pandemic—Anger	3968	1.48	1.43	0	4
Pandemic—Guilt	3967	0.38	0.82	0	4
Pandemic—Desperation	3970	1.62	1.40	0	4
Pandemic—Impact on psychosocial functioning	3975	1.04	1.06	0	3
Distress—Individual stressors	3998	9.11	6.06	0	24
Self-efficacy	3935	3.45	0.89	1	5
Expressive flexibility	3887	13.03	5.08	0	24
HRQoL	3916	45.57	11.10	0	84
Depression	3932	1.01	0.76	0	3
Anxiety	3954	1.03	0.56	0	2

*SD* standard deviation, *Min* minimum, *Max* maximum, *SES* socioeconomic Status, *HRQoL* health-related quality of life

### Depressive symptoms

**Fig. 1:**
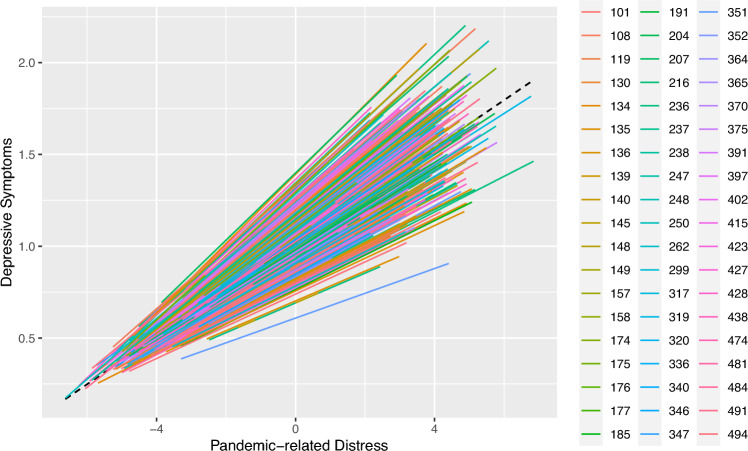
Association between pandemic-related distress and depressive symptoms. Each line represents an individual class and each colour an individual school

Observations were non-independent as reflected in an intraclass correlation coefficient (ICC) of 0.05. The model including crisis-related distress fitted our data significantly better than the intercept-only model (*χ*^2^diff(3) = 759.84, *p* < 0.001). Including a random slope for Pandemic-related distress further improved model fit (*χ*^2^diff (2) = 19.87, *p* < 0.001). Variance in slopes is illustrated in Fig. 1.

Including random slopes for war-related and climate-related distress did not result in further improvements in model fit (*p* > 0.05). Including socio-demographic variables further improved model fit (*χ*^2^diff (4) = 307.65, *p* < 0.001) as did individual stressor-related distress (*χ*^2^diff (1) = 760.37, *p* < 0.001) and resilience factors (*χ*^2^diff (2) = 216.48, *p* < 0.001). Table 2 provides an overview of intercepts and regression weights as well as estimated variance accounted for by fixed and random effects in each model.

**Table 2:** Model summaries of linear mixed model analyses for depressive symptoms

Predictors	Intercept only			Baseline model			+ RE distress—pandemic^+^		
	*B*	CI	*p*	*B*	CI	*p*	*B*	CI	*p*
Intercept	1.02	0.99–1.05	** < 0.001**	1.02	0.99–1.06	** < 0.001**	1.02	0.99–1.06	** < 0.001**
Distress—pandemic				0.11	0.10–0.12	** < 0.001**	0.11	0.10–0.12	** < 0.001**
Distress—war				0.00	− 0.01–0.02	0.520	0.00	− 0.01–0.02	0.478
Distress—climate				0.05	0.04–0.06	** < 0.001**	0.05	0.04–0.06	** < 0.001**
Age									
Ses									
Sex—female									
Sex—diverse									
Distress—individual									
Self-efficacy									
Expressive flexibility									
Random effects									
*σ*^2^	0.55			0.44			0.43		
τ_00_	0.03_School:Class_			0.04_School:Class_			0.04_School:Class_		
	0.00_School_			0.00_School_			0.00_School_		
τ_11_							0.00_School:Class.PANDEMIC_		
ρ_01_							0.84_School:Class_		
ICC	0.05			0.09			0.11		
N	57_School_			57_School_			57_School_		
	445_Class_			445_Class_			445_Class_		
Observations	3603			3603			3603		
Marginal *R*^2^/conditional *R*^2^	0.000/0.049			0.176/0.248			0.175/0.264		

Predictors	+ FE sociodemographics			+ FE distress—individual			+ FE resilience factors		
	*B*	CI	*p*	*B*	CI	*p*	*B*	CI	*p*
Intercept	1.03	0.99–1.06	** < 0.001**	1.03	0.99–1.06	** < 0.001**	1.03	0.99–1.06	** < 0.001**
Distress—pandemic	0.09	0.08–0.10	** < 0.001**	0.05	0.04–0.06	** < 0.001**	0.05	0.04–0.06	** < 0.001**
Distress—war	− 0.00	− 0.01–0.01	0.974	− 0.01	− 0.02– 0.00	0.204	− 0.00	– 0.01–0.01	0.427
Distress—climate	0.04	0.03–0.05	** < 0.001**	0.03	0.02–0.04	** < 0.001**	0.03	0.02–0.04	** < 0.001**
Age	0.04	− 0.00–0.08	0.071	0.03	− 0.01–0.06	0.146	0.03	− 0.01–0.07	0.103
Ses	– 0.05	− 0.06– – 0.03	** < 0.001**	-0.03	− 0.04–− 0.01	** < 0.001**	− 0.02	− 0.03–− 0.01	** < 0.001**
Sex—female	0.18	0.15–0.20	** < 0.001**	0.12	0.10–0.15	** < 0.001**	0.10	0.08–0.12	** < 0.001**
Sex—diverse	0.78	0.62–0.94	** < 0.001**	0.66	0.51–0.80	** < 0.001**	0.57	0.43–0.71	** < 0.001**
Distress—individual				0.06	0.05–0.06	** < 0.001**	0.05	0.05–0.05	** < 0.001**
Self-efficacy							− 0.17	– 0.19–− 0.15	** < 0.001**
Expressive flexibility							− 0.00	– 0.01– − 0.00	**0.018**
Random effects									
*σ*^2^	0.39			0.30			0.28		
τ_00_	0.05_School:Class_			0.06_School:Class_			0.07_School:Class_		
	0.00_School_			0.00_School_			0.00_School_		
τ_11_	0.00_School:Class.PANDEMIC_			0.00_School:Class.PANDEMIC_			0.00_School:Class.PANDEMIC_		
ρ_01_	0.73_School:Class_			0.62_School:Class_			0.56_School:Class_		
ICC	0.13			0.20			0.22		
N	57_School_			57_School_			57_School_		
	445_Class_			445_Class_			445_Class_		
Observations	3603			3603			3603		
Marginal *R*^2^/conditional *R*^2^	0.236/0.338			0.360/0.485			0.390/0.523		

*RE* random effect, *FE* fixed effect(s), *B* unstandardized regression weight, *CI* confidence Interval, *p* significance level, *Ses* socioeconomic Status, *ICC* intraclass correlation

^+^Including the school-level random slope for Pandemic-related distress resulted in model non-convergence due to extremely low variance in slopes between schools. Hence, we only included the class-level random slope in all subsequent models

Significant results (*p* < 0.05) are presented in bold

In the final model, fixed effects were estimated to account for 39% of the variance in depressive symptoms. Distress related to individual stressors was found to be the strongest predictor, reflecting that participants with higher distress ratings reported more symptoms [*β* = 0.36, standard error (SE) = 0.01, *p* < 0.001]. The next highest predictor was self-efficacy, which predicted fewer symptoms (*β* = – 0.18, SE = 0.01, *p* < 0.001). Pandemic-related distress (*β* = 0.15, SE = 0.02, *p* < 0.001), female sex (*β* = 0.12, SE = 0.01, *p* < 0.001), and diverse sex (*β* = 0.10, SE = 0.01, *p* < 0.001), Climate-related distress (*β* = 0.09, SE = 0.01, *p* < 0.001) were found to be related to higher symptoms. Finally, higher socio-economic status (*β* = − 0.04, SE = 0.01, *p* < 0.001) and higher expressive flexibility (*β* = − 0.03, SE = 0.01, *p* = 0.018) were found to be linked to fewer symptoms.

### Anxiety symptoms

Observations were non-independent as reflected in an ICC of 0.06. The model including crisis-related distress fitted our data significantly better than the intercept-only model (*χ*^2^diff(3) = 998.84, *p* < 0.001). Including random slopes for pandemic-related, war-related, and climate-related distress did not result in further improvements in model fit (*p* > 0.05). Including socio-demographic variables further improved model fit (*χ*^2^diff (4) = 346.58, *p* < 0.001) as did introduce individual stressor-related distress (*χ*^2^diff (1) = 458.92, *p* < 0.001) and resilience factors (*χ*^2^diff (2) = 188.78, *p* < 0.001). Table 3 provides an overview of intercepts and regression weights as well as estimated variance accounted for by fixed and random effects in each model.

**Table 3:** Model summaries of linear mixed model analyses for anxiety symptoms

Predictors	Intercept only^+^				Baseline model				+ FE sociodem				+ FE distress–individual					+ FE resilience factors		
	*B*		CI	*p*	*B*		CI	*p*	*B*		CI	*p*	*B*		CI	*p*		*B*	CI	*p*
Intercept	1.03		1.01–1.05	** < 0.001**	1.03		1.01–1.05	** < 0.001**	1.03		1.01–1.05	** < 0.001**	1.03		1.01–1.05	** < 0.001**		1.03	1.01–1.05	** < 0.001**
Distress—pandemic					0.08		0.07–0.08	** < 0.001**	0.06		0.06–0.07	** < 0.001**	0.04		0.03–0.05	** < 0.001**		0.04	0.03–0.04	** < 0.001**
Distress—war					0.02		0.02–0.03	** < 0.001**	0.02		0.01–0.03	** < 0.001**	0.02		0.01–0.02	** < 0.001**		0.02	0.01–0.02	** < 0.001**
Distress—climate					0.04		0.03–0.05	** < 0.001**	0.03		0.03–0.04	** < 0.001**	0.03		0.02–0.03	** < 0.001**		0.03	0.02–0.03	** < 0.001**
Age									0.01		− 0.02–0.04	0.469	0.01		− 0.02–0.03	0.707		0.00	− 0.02–0.03	0.726
Ses									− 0.02		− 0.03–− 0.01	** < 0.001**	− 0.01		− 0.02–− 0.00	**0.039**		− 0.01	− 0.02–0.00	0.155
Sex—female									0.16		0.15–0.18	** < 0.001**	0.14		0.12–0.15	** < 0.001**		0.12	0.10–0.13	** < 0.001**
Sex—diverse									0.16		0.05–0.27	**0.006**	0.09		− 0.01–0.20	0.088		0.04	− 0.06–0.14	0.446
Distress—individual													0.03		0.03–0.03	** < 0.001**		0.03	0.02–0.03	** < 0.001**
Self-efficacy																		− 0.12	− 0.14–− 0.11	** < 0.001**
Expressive flexibility																		0.00	0.00–0.01	**0.012**
Random effects																				
*σ*^2^		0.29				0.22				0.19				0.17			0.16			
τ_00_		0.02_School:Class_				0.03_School:Class_				0.03_School:Class_				0.04_School:Class_			0.04_School:Class_			
ICC		0.06				0.12				0.15				0.18			0.20			
N		57_School_				57_School_				57_School_				57_School_			57_School_			
		446_Class_				446_Class_				446_Class_				446_Class_			446_Class_			
Observations		3624				3624				3624				3624			3624			
Marginal *R*^2^/conditional *R*^2^		0.000/0.065				0.222/0.316				0.283/0.387				0.355/0.472			0.380/0.503			

*FE* fixed effect(s), *B* unstandardized regression weight, *CI* confidence Interval, *p* significance level, *Ses* socioeconomic status, *ICC* intraclass correlation

^+^For most models, including the school-level random intercept resulted in non-convergence due to extremely low variance in intercepts between schools. Hence, we only included the class-level random intercept in all models

Significant results (*p* < 0.05) are presented in bold

In the final model, fixed effects were estimated to account for 38% of the variance in anxiety symptoms. Distress related to individual stressors was found to be the strongest predictor, reflecting that participants with higher distress ratings reported more symptoms (*β* = 0.27, SE = 0.01, *p* < 0.001). The next highest predictors were female sex (*β* = 0.18, SE = 0.01, *p* < 0.001) and self-efficacy (*β* = − 0.18, SE = 0.01, *p* < 0.001), which predicted more and fewer symptoms, respectively. Pandemic-related distress (*β* = 0.15, SE = 0.01, *p* < 0.001), climate-related distress (*β* = 0.10, SE = 0.01, *p* < 0.001), and war-related distress (*β* = 0.07, SE = 0.01, *p* < 0.001) were found to be related to higher symptoms. Finally, contrary to our assumption, higher expressive flexibility was found to be linked to higher symptoms (*β* = 0.03, SE = 0.01, *p* < 0.001).

### HRQoL

**Fig. 2:**
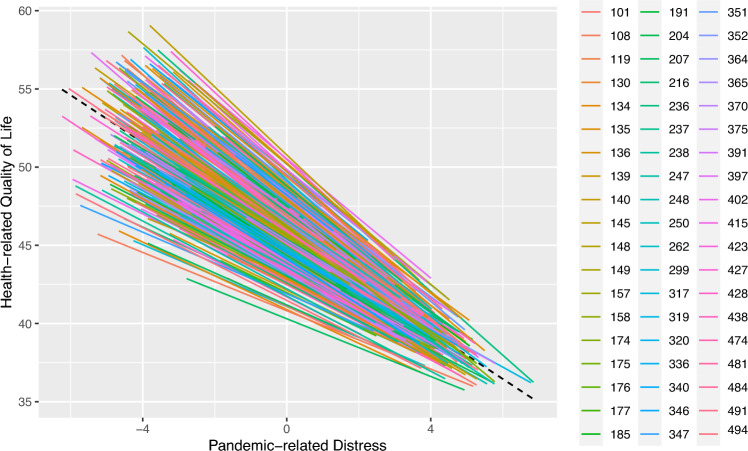
Association between Pandemic-related distress and HrQoL. Each line represents an individual class and each colour an individual school

Observations were non-independent as reflected in an ICC of 0.05. The model including crisis-related distress fitted our data significantly better than the intercept-only model (χ^2^diff(3) = 467.20, *p* < 0.001). Including a random slope for Pandemic-related distress further improved model fit (χ^2^diff (4) = 11.46, *p* = 0.022). Variance in slopes is illustrated in Fig. 2.

Including random slopes for war-related and climate-related distress did not result in further improvements in model fit (*p* > 0.05). Including socio-demographic variables further improved model fit (χ^2^diff (4) = 271.50, *p* < 0.001) as did introduce individual stressor-related distress (χ^2^diff (1) = 575.19, *p* < 0.001) and resilience factors (χ^2^diff (2) = 496.99, *p* < 0.001). Table 4 provides an overview of intercepts and regression weights as well as estimated variance accounted for by fixed and random effects in each model.

**Table 4:** Model summaries of linear mixed model analyses for Health-related quality of life

Predictors		Intercept only				Baseline model				+ RE distress—pandemic		
		*B*	CI	*p*		*B*	CI	*p*		*B*	CI	*p*
(Intercept)		45.34	44.77–45.92	** < 0.001**		45.33	44.75–45.90	** < 0.001**		45.34	44.76–45.91	** < 0.001**
Distress—pandemic						− 1.26	− 1.42–− 1.10	** < 0.001**		− 1.27	− 1.44–− 1.09	** < 0.001**
Distress—war						− 0.04	− 0.21–0.13	0.653		− 0.04	− 0.22–0.13	0.624
Distress—climate						− 0.59	− 0.77–− 0.42	** < 0.001**		− 0.59	− 0.76–− 0.41	** < 0.001**
Age												
Ses												
Sex—female												
Sex—diverse												
Distress—individual												
Self-efficacy												
Expressive flexibility												
Random effects												
*σ*^2^	116.39				100.67				98.54			
τ_00_	4.91_School:Class_				6.71_School:Class_				7.14_School:Class_			
	1.80_School_				1.86_School_				1.82_School_			
τ_11_									0.33_School:Class.PANDEMIC_			
									0.01_School.PANDEMIC_			
ρ_01_									− 0.56_School:Class_			
									0.30_School_			
ICC	0.05				0.08				0.10			
N	57_School_				57_School_				57_School_			
	447_Class_				447_Class_				447_Class_			
Observations	3595				3595				3595			
Marginal *R*^2^/conditional *R*^2^	0.000/0.054				0.114/0.184				0.115/0.202			

Predictors		+ FE sociodemographics				+ FE distress – individual^+^				+ FE resilience factors		
		*B*	CI	*p*		*B*	CI	*p*		*B*	CI	*p*
(Intercept)		45.34	44.78–45.90	** < 0.001**		45.31	44.74–45.88	** < 0.001**		45.31	44.74–45.88	** < 0.001**
Distress—pandemic		− 1.05	− 1.22–− 0.88	** < 0.001**		− 0.49	− 0.65–− 0.33	** < 0.001**		− 0.36	− 0.51–− 0.20	** < 0.001**
Distress—war		0.03	− 0.14–0.19	0.746		0.10	− 0.05–0.25	0.182		0.04	− 0.10–0.18	0.593
Distress—climate		− 0.48	− 0.65–− 0.31	** < 0.001**		− 0.31	− 0.46–− 0.16	** < 0.001**		− 0.31	− 0.46–− 0.17	** < 0.001**
Age		− 0.42	− 1.04–0.20	0.186		− 0.25	− 0.82–0.33	0.398		− 0.32	− 0.85–0.21	0.237
Ses		0.94	0.73–1.15	** < 0.001**		0.63	0.43–0.82	** < 0.001**		0.53	0.35–0.71	** < 0.001**
Sex—female		− 2.70	− 3.08–− 2.32	** < 0.001**		− 2.01	− 2.36–− 1.66	** < 0.001**		− 1.50	− 1.83–− 1.17	** < 0.001**
Sex—diverse		− 5.95	− 8.42–− 3.47	** < 0.001**		− 4.19	− 6.46–− 1.93	** < 0.001**		− 2.23	− 4.33–− 0.13	**0.037**
Distress—individual						− 0.76	− 0.82–− 0.70	** < 0.001**		− 0.61	− 0.67–− 0.56	** < 0.001**
Self-efficacy										3.84	3.48–4.19	** < 0.001**
Expressive flexibility										0.16	0.10–0.22	** < 0.001**
Random effects												
*σ*^2^	90.34				75.70				64.33			
τ_00_	8.31_School:Class_				10.52_School:Class_				12.76_School:Class_			
	1.62_School_				1.69_School_				1.61_School_			
τ_11_	0.36_School:Class.PANDEMIC_				0.23_School:Class.PANDEMIC_				0.20_School:Class.PANDEMIC_			
	0.00_School.PANDEMIC_								0.03_School.PANDEMIC_			
ρ_01_	− 0.47_School:Class_				− 0.61_School:Class_				− 0.56_School:Class_			
	0.75_School_								0.30_School_			
ICC	0.12				0.15				0.20			
N	57_School_				57_School_				57_School_			
	447_Class_				447_Class_				447_Class_			
Observations	3595				3595				3595			
Marginal *R*^2^/conditional *R*^2^	0.173/0.270				0.281/0.390				0.359/0.484			

*RE* random effect, *FE* fixed effect(s), *B* unstandardized regression weight, *CI* confidence Interval, *p* significance level, *Ses* socioeconomic Status, *ICC* intraclass correlation

^+^Including the school-level random slope for Pandemic-related distress resulted in non-convergence due to extremely low variance in slopes between schools. Since this issue occurred exclusively in this specific submodel we excluded the school-level slope only in this model

Significant results (*p* < 0.05) are presented in bold

In the final model, fixed effects were estimated to account for 35.9% of the variance in HRQoL. Distress related to individual stressors was found to be the strongest predictor, reflecting that participants with higher distress ratings reported lower HRQoL (*β* = − 0.30, SE = 0.01, *p* < 0.001). The next highest predictor was self-efficacy, which predicted higher HRQoL (*β* = 0.28, SE = 0.01, *p* < 0.001). Female sex (*β* = − 0.12, SE = 0.01, *p* < 0.001) and Pandemic-related distress (*β* = − 0.08, SE = 0.02, *p* < 0.001) were found to be related to higher symptoms. Higher socio-economic status (*β* = 0.07, SE = 0.01, *p* < 0.001) and expressive flexibility (*β* = 0.07, SE = 0.01, *p* < 0.001) were linked to higher HRQoL whereas higher Climate-related distress (*β* = − 0.06, SE = 0.01, *p* < 0.001) and diverse sex (*β* = − 0.03, SE = 0.01, *p* = 0.037) were associated with lower HRQoL.

## Discussion

The present study investigated the impact of current global crises on adolescents´ mental health in Germany. In line with assumptions, we found that crises-related distress was associated with greater depression and anxiety as well as lower HRQoL. Effects were consistently evident for pandemic- and climate-related distress. For war-related distress, effects only emerged for anxiety symptoms. Controlling for socio-demographic factors, individual life stressors, and resilience factors did not affect the significance of these effects, confirming incremental predictive power. Fixed effects accounted for 36–39% of variance in symptoms in the final models.

Overall, these findings demonstrate the significant impact of global crises on adolescents´ mental health: Taking only crises-related distress into account, we were able to predict 11–22% variance in symptoms. While previous studies have demonstrated significant effects of individual crises [5, 10, 13, 38], this study is the first to show their simultaneous impact on adolescents. Our findings align with Barchielli et al. [16], who found that all three global crises predicted mental health in Italian adults. They found that climate-related stress was the strongest predictor of depression, while pandemic-related stress was the strongest predictor of anxiety. By contrast, our study shows that pandemic-related distress was the strongest predictor across all outcome measures. In this respect, our study converges with the findings of an adult sample in Germany [15] that revealed that pandemic-related distress, but not war- or climate-related distress, significantly predicts mental health.

Our finding that pandemic-related distress had the strongest impact on symptoms may be due to the circumstance that the pandemic had a significant impact on the daily lives of young people in the 2.5 years prior to assessment [7]. Given that the daily consequences of the climate crisis and the RUW are less marked for adolescents living in Germany, it seems plausible that pandemic-related stress was the strongest predictor of mental health at measurement time. This assumption is further supported by the fact that war-related distress was the weakest predictor since this crisis had only emerged shortly before our assessment period. Further research needs to reassess how these effects change with time and the course of the respective crises. Therefore, we plan further survey dates in the future.

A great strength of the current study is that it established incremental predictive value by controlling for several established risk and resilience factors. In line with previous research, our data show that several sociodemographic characteristics (lower socioeconomic status, female gender, and diverse gender) were linked to greater symptom load [39]. Moreover, distress related to individual stressors was found to be the strongest predictor across all analyses. This is in line with literature demonstrating that adverse life experiences are among the strongest predictors of mental health in adolescents [40–42]. Finally, resilience factors were found to account for additional variance in symptoms, with self-efficacy having a greater impact than expressive flexibility. While found effects generally indicated that greater self-efficacy and expressive flexibility were linked to fewer symptoms/higher HRQoL, expressive flexibility was surprisingly found to predict higher anxiety. Although unexpected, previous research on college students has shown that higher flexibility is linked to fewer depression symptoms but higher post-traumatic stress disorder symptoms [43]. The authors interpret this finding as indicating that very high levels of emotional flexibility may in fact indicate emotional avoidance and that the concurrent lack of emotional processing may in turn be linked to higher symptom load [44, 45].

### Limitations

Several limitations of our study should be noted. First, the study was cross-sectional, which does not allow us to draw causal inference. Moreover, while we were able to establish a link between global crises and mental health, the study cannot shed light on underlying mechanisms. Future longitudinal studies should aim to address these gaps by assessing potential mediators (e.g. future thinking; [46]). Another limitation is that our study did not use a probabilistic sampling strategy, which limits the generalization of our findings to the wider population of adolescents. A further restriction is the mixed-mode design of our study: For feasibility reasons, participants were able to respond either online or by paper–pencil, which may have introduced additional noise. Finally, our study relied on self-assessments only, which limits the validity of symptom measurement. Despite these limitations, it is important to consider the strengths of the current sample, particularly its comprehensive size and its sociodemographic diversity (due to school-based assessment).

## Conclusion

Overall, our findings illustrate the strong impact of global crises on adolescents’ mental health. All crises affected mental health, albeit to a different extent. COVID-19 had the strongest effect, the climate crisis had a weaker, yet consistent, effect, and RUW had the weakest effect, which was limited to anxiety symptoms. Critically, all these effects remained significant after controlling for several established covariates, thereby suggesting that global crises constitute an independent predictor of mental health. Thus, the current results suggest that future research should further quantify the impacts of global crises on mental health and the effectiveness of mitigation strategies in dealing with the crises. Moreover, they suggest that policy responses should include interventions fostering resilience and support adolescents in coping with crises-related distress [47]. Such interventions should aim to enhance self-efficacy and other resilience factors indirectly by training transdiagnostic factors such as stress management and emotion regulation skills (e.g. [48]).

### Supplementary Information

Below is the link to the electronic supplementary material.

Supplementary file1 (DOCX 29 KB)

## Data Availability

The data that support the findings of this study are available from the corresponding author upon reasonable request.

## Abbreviations

COVID-19: Coronavirus disease 2019
RUW: Russia-Ukraine War
HRQol: Health-related quality of life
CAFE scale: Child and adolescent flexible expressiveness scale
SE: Standard error

## Footnotes

1 The CAFE scale comprises two subscales assessing the ability to enhance and suppress emotional expressions. A flexibility score was calculated based on these subscales. In addition, we conducted follow-up analyses including both subscale scores as independent predictors into the final model structure (see Supplementary Information).

## References

[1] Heading S, Zahidi S (2023). Global risks report 2023.

[2] Kikstra JS, Nicholls ZRJ, Smith CJ, Lewis J, Lamboll RD, Byers E, Sandstad M, Meinshausen M, Gidden MJ, Rogelj J, Kriegler E, Peters GP, Fuglestvedt JS, Skeie RB, Samset BH, Wienpahl L, van Vuuren DP, van der Wijst K-I, Al Khourdajie A, Forster PM, Reisinger A, Schaeffer R, Riahi K (2022). The IPCC sixth assessment report WGIII climate assessment of mitigation pathways: from emissions to global temperatures. Geosci Model Dev.

[3] Solmi M, Radua J, Olivola M, Croce E, Soardo L, Salazar de Pablo G, Il Shin J, Kirkbride JB, Jones P, Kim JH, Kim JY, Carvalho AF, Seeman MV, Correll CU, Fusar-Poli P (2022). Age at onset of mental disorders worldwide: large-scale meta-analysis of 192 epidemiological studies. Mol Psychiatry.

[4] Patel V, Saxena S, Lund C, Thornicroft G, Baingana F, Bolton P, Chisholm D, Collins PY, Cooper JL, Eaton J, Herrman H, Herzallah MM, Huang Y, Jordans MJD, Kleinman A, Medina-Mora ME, Morgan E, Niaz U, Omigbodun O, Prince M, Rahman A, Saraceno B, Sarkar BK, De Silva M, Singh I, Stein DJ, Sunkel C, Jü UnÜtzer (2018). The lancet commission on global mental health and sustainable development. Lancet.

[5] Andresen S, Heyer L, Lips A, Rusack T, Schröer W, Thomas S, Wilmes J (2020). “Die Corona-Pandemie hat mir wertvolle Zeit genommen”—Jugendalltag 2020.

[6] Pieh C, Plener PL, Probst T, Dale R, Humer E (2021). Assessment of mental health of high school students during social distancing and remote schooling during the COVID-19 pandemic in Austria. JAMA Netw Open.

[7] Ravens-Sieberer U, Erhart M, Devine J, Gilbert M, Reiss F, Barkmann C, Siegel NA, Simon AM, Hurrelmann K, Schlack R, Hölling H, Wieler LH, Kaman A (2022). Child and adolescent mental health during the COVID-19 pandemic: results of the three-wave longitudinal COPSY Study. J Adolesc Health.

[8] Thorisdottir IE, Agustsson G, Oskarsdottir SY, Kristjansson AL, Asgeirsdottir BB, Sigfusdottir ID, Valdimarsdottir HB, Allegrante JP, Halldorsdottir T (2023). Effect of the COVID-19 pandemic on adolescent mental health and substance use up to March, 2022, in Iceland: a repeated, cross-sectional, population-based study. Lancet Child Adolesc Health.

[9] Möller-Slawinski H (2021) Ergebnisse einer Repräsentativ-Umfrage unter Jugendlichen Eine SINUS-Studie im Auftrag der BARMER

[10] Hickman C, Marks E, Pihkala P, Clayton S, Lewandowski RE, Mayall EE, Wray B, Mellor C, van Susteren L (2021). Climate anxiety in children and young people and their beliefs about government responses to climate change: a global survey. Lancet Planet Health.

[11] Ogunbode CA, Pallesen S, Böhm G, Doran R, Bhullar N, Aquino S, Marot T, Schermer JA, Wlodarczyk A, Lu S, Jiang F, Salmela-Aro K, Hanss D, Maran DA, Ardi R, Chegeni R, Tahir H, Ghanbarian E, Park J, Tsubakita T, Tan C-S, van den Broek KL, Chukwuorji JC, Ojewumi K, Reyes MES, Lins S, Enea V, Volkodav T, Sollar T, Navarro-Carrillo G, Torres-Marín J, Mbungu W, Onyutha C, Lomas MJ (2023). Negative emotions about climate change are related to insomnia symptoms and mental health: cross-sectional evidence from 25 countries. Curr Psychol.

[12] Ojala M, Cunsolo A, Ogunbode CA, Middleton J (2021). Anxiety, worry, and grief in a time of environmental and climate crisis: a narrative review. Annu Rev Environ Resour.

[13] Riad A, Dobrov A, Krobot M, Antalová N, Alkasaby MA, Peřina A, Koščík M (2022). Mental health burden of the Russian-Ukrainian war 2022 (RUW-22): anxiety and depression levels among young adults in central Europe. Int J Environ Res Public Health.

[14] Runze J, Marten F, TeBrinke L (2022). The effect of exposure to social-media coverage of the Russo-Ukrainian war on stress symptoms in Dutch adolescents.

[15] Weierstall-Pust R, Schnell T, Heßmann P, Feld M, Höfer M, Plate A, Müller MJ (2022). Stressors related to the COVID-19 pandemic, climate change, and the Ukraine crisis, and their impact on stress symptoms in Germany: analysis of cross-sectional survey data. BMC Public Health.

[16] Barchielli B, Cricenti C, Gallè F, Sabella EA, Liguori F, Da Molin G, Liguori G, Orsi GB, Giannini AM, Ferracuti S, Napoli C (2022). Climate changes, natural resources depletion, COVID-19 Pandemic, and Russian-Ukrainian war: what is the impact on habits change and mental health?. Int J Environ Res Public Health.

[17] Bandura A, Freeman WH, Lightsey R (1999). Self-efficacy: the exercise of control. J Cogn Psychother.

[18] Scheiner C, Seis C, Kleindienst N, Buerger A (2023). Psychopathology, protective factors, and COVID-19 among adolescents: a structural equation model. Int J Environ Res Public Health.

[19] Maran DA, Begotti T (2021). Media exposure to climate change, anxiety, and efficacy beliefs in a sample of Italian university students. Int J Environ Res Public Health.

[20] Bonanno GA (2021). The resilience paradox. Eur J Psychotraumatol.

[21] Hemi A, Sopp MR, Schäfer SK, Michael T, Levy-Gigi E (2022). Adaptive responding to prolonged stress exposure: a binational study on the impact of flexibility on latent profiles of cognitive, emotional and behavioral responses to the COVID-19 pandemic. Health Soc Care Community.

[22] Hemi A, Sopp MR, Bonanno G, Michael T, McGiffin J, Levy-Gigi E (2023). Flexibility predicts chronic anxiety and depression during the first year of the COVID-19 pandemic—A longitudinal investigation of mental health trajectories. Psychol Trauma Theory Res Pract Policy.

[23] Gruber J, Clark LA, Abramowitz JS, Aldao A, Chung T, Forbes EE, Nagayama Hall GC, Hinshaw SP, Hollon SD, Klein DN, Levenson RW, McKay D, Mendle J, Neblett EW, Olatunji BO, Prinstein MJ, Rottenberg J, Albano AM, Borelli JL, Davila J, Gee DG, Hallion LS, Hofmann SG, Joormann J, Kazdin AE, La Greca AM, MacDonald AW, McLaughlin KA, Miller AB, Nock M, Persons JB, Rozek DC, Slavich GM, Vine V, Schleider JL, Teachman BA, Weinstock LM (2021). Mental health and clinical psychological science in the time of COVID-19: challenges, opportunities, and a call to action. Am Psychol.

[24] Haag A-C, Cha CB, Noll JG, Gee DG, Shenk CE, Schreier HMC, Heim CM, Shalev I, Rose EJ, Jorgensen A, Bonanno GA (2023). The flexible regulation of emotional expression scale for youth (FREE-Y): adaptation and validation across a varied sample of children and adolescents. Psychol Assess.

[25] Wang Y, Hawk ST (2020). Development and validation of the child and adolescent flexible expressiveness (CAFE) Scale. Psychol Assess.

[26] Wang Y, Hawk ST (2020). Expressive enhancement, suppression, and flexibility in childhood and adolescence: longitudinal links with peer relations. Emotion.

[27] Fu F, Chow A, Li J, Cong Z (2018). Emotional flexibility: development and application of a scale in adolescent earthquake survivors. Psychol Trauma Theory Res Pract Policy.

[28] Fu F, Chow A (2017). Traumatic exposure and psychological well-being: the moderating role of cognitive flexibility. J Loss Trauma.

[29] Hoebel J, Müters S, Kuntz B, Lange C, Lampert T (2015). Messung des subjektiven sozialen Status in der Gesundheitsforschung mit einer deutschen Version der MacArthur Scale. Bundesgesundheitsblatt Gesundheitsforschung Gesundheitsschutz.

[30] Summers J, Winefield H (2009). Anxiety about war and terrorism in Australian high-school children. J Child Media.

[31] Nandakumar AL, Vande Voort JL, Nakonezny PA, Orth SS, Romanowicz M, Sonmez AI, Ward JA, Rackley SJ, Huxsahl JE, Croarkin PE (2019). Psychometric properties of the patient health questionnaire-9 modified for major depressive disorder in adolescents. J Child Adolesc Psychopharmacol.

[32] Weitkamp K, Romer G, Rosenthal S, Wiegand-Grefe S, Daniels J (2010). German screen for child anxiety related emotional disorders (SCARED): reliability, validity, and cross-informant agreement in a clinical sample. Child Adolesc Psychiatry Ment Health.

[33] Ravens-Sieberer U, Erhart M, Rajmil L, Herdman M, Auquier P, Bruil J, Power M, Duer W, Abel T, Czemy L, Mazur J, Czimbalmos A, Tountas Y, Hagquist C, Kilroe J, the European KIDSCREEN Group (2010). Reliability, construct and criterion validity of the KIDSCREEN-10 score: a short measure for children and adolescents’ well-being and health-related quality of life. Qual Life Res.

[34] Beierlein C, Kovaleva A, Kemper CJ, Rammstedt B (2012). Ein Messinstrument zur Erfassung subjektiver Kompetenzerwartungen.

[35] Bates DM (2010). lme4: mixed-effects modeling with R.

[36] Team RC (2022) R: A language and environment for statistical computing (version 4.2. 0) [Computer software] (4.2. 0). R Foundation for Statistical Computing

[37] Kreft IGG, de Leeuw J, Aiken LS (1995). The effect of different forms of centering in hierarchical linear models. Multivar Behav Res.

[38] Ravens-Sieberer U, Kaman A, Erhart M, Devine J, Schlack R, Otto C (2022). Impact of the COVID-19 pandemic on quality of life and mental health in children and adolescents in Germany. Eur Child Adolesc Psychiatry.

[39] Ravens-Sieberer U, Torsheim T, Hetland J, Vollebergh W, Cavallo F, Jericek H, Alikasifoglu M, Välimaa R, Ottova V, Erhart M (2009). Subjective health, symptom load and quality of life of children and adolescents in Europe. Int J Public Health.

[40] Ceccarelli C, Prina E, Muneghina O, Jordans M, Barker E, Miller K, Singh R, Acarturk C, Sorsdhal K, Cuijpers P, Lund C, Barbui C, Purgato M (2022). Adverse childhood experiences and global mental health: avenues to reduce the burden of child and adolescent mental disorders. Epidemiol Psychiatr Sci.

[41] Oh KS, Han JS (1990). Stressful life events, health symptoms, social support and coping in early adolescents. J Nurses Acad Soc.

[42] Roberts YH, English D, Thompson R, White CR (2018). The impact of childhood stressful life events on health and behavior in at-risk youth. Child Youth Serv Rev.

[43] Haim-Nachum S, Levy-Gigi E (2021). To be or not to be flexible: selective impairments as a means to differentiate between depression and PTSD symptoms. J Psychiatr Res.

[44] Sheppes G, Gross JJ, Weiner I (2012). Emotion regulation effectiveness: what works when. Handbook of Psychology.

[45] Wilson TD, Gilbert DT (2008). Explaining away: a model of affective adaptation. Perspect Psychol Sci.

[46] Poletti M, Preti A, Raballo A (2022). From economic crisis and climate change through COVID-19 pandemic to Ukraine war: a cumulative hit-wave on adolescent future thinking and mental well-being. Eur Child Adolesc Psychiatry.

[47] Zbukvic I, McKay S, Cooke S, Anderson R, Pilkington V, McGillivray L, Bailey A, Purcell R, Tye M (2023). Evidence for targeted and universal secondary school-based programs for anxiety and depression: an overview of systematic reviews. Adolesc Res Rev.

[48] Dawson KS, Watts S, Carswell K, Shehadeh MH, Jordans MJ, Bryant RA, Miller KE, Malik A, Brown FL, Servili C, van Ommeren M (2019). Improving access to evidence-based interventions for young adolescents: early adolescent skills for emotions (EASE). World Psychiatry.

