# Supplementary material for: Generation climate crisis, COVID-19, and Russia–Ukraine-War: global crises and mental health in adolescents
Source: Eur Child Adolesc Psychiatry. 2023 Oct 9;33(7):2203–16. doi: 10.1007/s00787-023-02300-x (PMC11255088; doi:10.1007/s00787-023-02300-x)
Supplement: Supplementary file 1 — Supplementary file1 (DOCX 29 KB) [file 787_2023_2300_MOESM1_ESM.docx]

**Supplementary Information**

*Generation climate crisis, COVID-19, and Russia-Ukaine-War– Global crises and mental health in adolescents*

___________________________________________________________________________

**Crisis-related distress**

Pandemic-related, war-related, and climate-related distress were assessed with self-developed questionnaires. As a basis for the questionnaires, we used previously established questionnaires on climate anxiety (Hickman et al. 2021), fear of war ( Summers & Winefield, 2009) and burden during the Covid-19 pandemic (Ravens-Sieberer, Erhart, et al., 2022).

The self-developed questionnaires consisted of the same questions for each crisis, which were answered on a 5-point Likert scale (perceived overall burden due to the crisis, affective reactions in response to the crisis, and impact of the crisis on psychosocial functioning). The majority of the items in our crises-related-questionnaires were adopted from previously established questionnaires on the different crisis. English items were translated into German by two individual translators. Furthermore, ten 7-9 graders pretested the entire questionnaire package and provided feedback on the clarity and wording of the questionnaire.

**Psychopathological Symptoms**

***Depression***

The Patient Health questionnaire (modified for Adolescent) (Nandakumar et al., 2019) was used to assess depressive symptoms on the basis of 9-items on a 4-point Likert scale (not at all - nearly every day). We calculated the mean response for all further analyses, allowing for a maximum of 20% missing responses.

***Generalized Anxiety***

The Generalized Anxiety subscale of the German version of the Screen for Child Anxiety Related Disorders (SCARED) (Weitkamp et al., 2010) consists of nine items (e.g., “I worry about what is going to happen in the future”) which are presented on a 3-point scale (0 = “not true or hardly ever true” to 2 = “very true or often true”). We calculated the man item rating for all further analyses, allowing for a maximum of 20% missing responses.

***Health related quality of Life***

The KIDSCREEN-10 Index (Ravens-Sieberer et al., 2010) assesses health-related quality of life including physical, psychological, and social aspects of quality of life in children and
adolescents from 8 to18 years old. It consists of ten items that are answered on a 5-point scale (0 = “never” to 4 = “always” or 0 = “not at all” to 4 = “extremely”). T-transformed Rasch person parameters were calculated for each participant based on the scoring instructions provided by the authors and allowing for a maximum of 10% missing responses (Ravens-Sieberer et al., 2010).

**Protective Factors**

***Self-efficacy***

Self-efficacy was assessed using the ASKU (*Allgemeine Selbstwirksamkeit Kurzskala*; Beierlein et al., 2012), which consists of 3 items measuring individual competence expectations in dealing with difficulties and obstacles in daily life (e.g. I am able to solve most problems on my own.) on a 5-point Likert scale. To obtain a global self-efficacy index, responses are averaged (range between 1 -5) with higher scores indicating higher self-efficacy.

***Expressive flexibility***

Six items of the child and adolescent flexible expressiveness scale (CAFE; Wang & Hawk, 2020a) were used to assess expressive flexibility. Due to time constraints, we could not include the complete scale. Thus, we chose the six items with the highest factor loading. The CAFE is a scenario-based questionnaire assessing the ability to flexibly suppress emotion expression or enhance emotion expression. Participants are asked to indicate their agreement on each item on a 5-point Likert scale. Enhancement and suppression scores were calculated by summing item scores of each subscale. A balanced expressive flexibility score was calculated by subtracting the absolute value of the difference between the two subscale scores from their sum, namely Expressive Flexibility = (Enhancement + Suppression) − |Enhancement – Suppression]. This formula assures that extremely high scores on only one form of expressive regulation, but not the other, would still yield lower overall scores.

**Informed Consent**

A member of our research team visited each participating school and informed pupils about the study. A detailed study information and an informed consent form was handed out to each pupil (pupil and parent version).

**Onsite Assessment**

Two members of our research team visited each school for assessment. Pupils, who had returned the informed consent forms, and wished to participate filled out the questionnaire package (online version or paper pencil version) during one school lesson. Completion took about 25-40 minutes. During assessment, a member of the research team was present to answer questions regarding the questionnaire. For details on data entry see Supplementary Information.

**Data entry**

For the online version of the questionnaire package, measures were collected via the online platform SoSci Survey (Leiner, 2019). Data entry of the paper-pencil questionnaires was conducted separately by two different student assistants in order to control for possible data entry errors. If there was a mismatch between the two versions, a member of the research team checked the items in the original questionnaire and corrected each error. Finally, online data and paper pencil data were integrated into one data set.

**Table S1.** Model summaries of analyses including the CAFE subscales as independent predictors.

|  | **DEPRESSION** | | | **ANXIETY** | | | **HRQoL** | | |
| --- | --- | --- | --- | --- | --- | --- | --- | --- | --- |
| *Predictors* | *B* | *CI* | *p* | *B* | *CI* | *p* | *B* | *CI* | *p* |
| (Intercept) | 1.03 | 0.99 – 1.06 | **<0.001** | 1.03 | 1.01 – 1.05 | **<0.001** | 45.31 | 44.75 – 45.88 | **<0.001** |
| Distress - Pandemic | 0.05 | 0.04 – 0.06 | **<0.001** | 0.04 | 0.03 – 0.04 | **<0.001** | -0.35 | -0.51 – -0.19 | **<0.001** |
| Distress - War | -0.00 | -0.01 – 0.00 | 0.364 | 0.02 | 0.01 – 0.02 | **<0.001** | 0.04 | -0.10 – 0.18 | 0.558 |
| Distress - Climate | 0.03 | 0.02 – 0.04 | **<0.001** | 0.03 | 0.02 – 0.03 | **<0.001** | -0.31 | -0.46 – -0.17 | **<0.001** |
| Age | 0.03 | -0.00 – 0.07 | 0.087 | 0.01 | -0.02 – 0.03 | 0.665 | -0.33 | -0.86 – 0.20 | 0.226 |
| Ses | -0.02 | -0.03 – -0.01 | **<0.001** | -0.01 | -0.02 – 0.00 | 0.158 | 0.53 | 0.35 – 0.71 | **<0.001** |
| Sex - Female | 0.09 | 0.07 – 0.12 | **<0.001** | 0.11 | 0.10 – 0.13 | **<0.001** | -1.48 | -1.81 – -1.15 | **<0.001** |
| Sex - Diverse | 0.57 | 0.43 – 0.71 | **<0.001** | 0.04 | -0.06 – 0.14 | 0.447 | -2.21 | -4.31 – -0.11 | **0.039** |
| Distress - Individual | 0.05 | 0.05 – 0.05 | **<0.001** | 0.03 | 0.02 – 0.03 | **<0.001** | -0.61 | -0.67 – -0.55 | **<0.001** |
| Self-Efficacy | -0.17 | -0.20 – -0.15 | **<0.001** | -0.12 | -0.14 – -0.11 | **<0.001** | 3.84 | 3.49 – 4.20 | **<0.001** |
| Enhancement subscale | 0.02 | 0.01 – 0.03 | **<0.001** | 0.02 | 0.01 – 0.02 | **<0.001** | 0.06 | -0.07 – 0.18 | 0.362 |
| Suppressing subscale | -0.02 | -0.02 – -0.01 | **<0.001** | -0.00 | -0.01 – 0.00 | 0.262 | 0.24 | 0.13 – 0.36 | **<0.001** |
| **Random Effects** | | | | | | | | | |
| σ^2^ | 0.28 | | | 0.16 | | | 64.53 | | |
| τ_00_ | 0.07 _School:Class_ | | | 0.04 _School:Class_ | | | 12.72 _School:Class_ | | |
|  | 0.00 _School_ | | |  | | | 1.60 _School_ | | |
| τ_11_ | 0.00 _School:Class.PANDEMIC_ | | |  | | | 0.18 _School:Class.PANDEMIC_ | | |
|  |  | | |  | | | 0.04 _School.PANDEMIC_ | | |
| ρ_01_ | 0.56 _School:Class_ | | |  | | | -0.58 _School:Class_ | | |
|  |  | | |  | | | 0.31 _School_ | | |
| ICC | 0.22 | | | 0.20 | | | 0.19 | | |
| N | 57 _School_ | | | 57 _School_ | | | 57 _School_ | | |
|  | 445 _Class_ | | | 446 _Class_ | | | 447 _Class_ | | |
| Observations | 3603 | | | 3624 | | | 3595 | | |
| Marginal R^2^ / Conditional R^2^ | 0.392 / 0.526 | | | 0.383 / 0.507 | | | 0.358 / 0.483 | | |

*Note:* CAFE = Child and Adolescent Flexible Expressiveness Scale, HRQoL = Health-related quality of life, RE = Random effect, FE = Fixed effect(s), B = unstandardized regression weight, CI = Confidence Interval, p = significance level, Ses = Socioeconomic Status, ICC = Intraclass correlation.
